# Utility of flow cytometry screening before MRD testing in multiple myeloma

**DOI:** 10.1038/s41408-023-00832-8

**Published:** 2023-04-20

**Authors:** Vandana Panakkal, Arjun Lakshman, Min Shi, Horatiu Olteanu, Pedro Horna, Michael M. Timm, Gregory E. Otteson, Linda B. Baughn, Patricia T. Greipp, Wilson I. Gonsalves, Prashant Kapoor, Morie A. Gertz, Moritz Binder, Francis K. Buadi, Angela Dispenzieri, S. Vincent Rajkumar, Shaji K. Kumar, Dragan Jevremovic

**Affiliations:** 1 Division of Hematology, Department of Medicine, Mayo Clinic, Rochester, MN USA; 2 Department of Medical Oncology, Mayo Clinic, Rochester, MN USA; 3 Division of Hematopathology, Mayo Clinic, Rochester, MN USA; 4 Division of Laboratory Genetics and Genomics, Department of Laboratory Medicine and Pathology, Mayo Clinic, Rochester, MN USA

**Keywords:** Myeloma, Myeloma

**TO THE EDITOR:**

Multiple myeloma (MM) is the second most common hematological malignancy in the United States predicted to cause 34,470 new cases and 12,640 deaths in 2022 [1]. Outcomes of patients with MM continue to improve with the advent of highly effective multidrug therapy regimens and high-dose chemotherapy with autologous stem cell transplant [2]. With improvements in flow cytometry (FCM) and next-generation sequencing (NGS) technologies, attaining measurable residual disease (MRD) negativity in the bone marrow (BM) after treatment, a deeper level of response than stringent complete response (sCR), has emerged as an important prognostic factor for patients [3]. Next-generation flow cytometry (NGF) and NGS are the recommended techniques for MRD detection currently in use [4]. The accepted NGF MRD method is a 2-tube, 10-antibody (Ab) test (Euroflow Consortium) with a minimum sensitivity of 10^−5^ [5, 6]. This assay requires significant processing, instruments, and analysis time [6].

In practice, clinical response assessment is often incomplete at the time of BM evaluation, as the results of serum and urine monoclonal protein studies and advanced imaging are not available due to the logistics of patient scheduling and test turnaround time. With the paucity of the above data, in most cases, sCR cannot be ascertained prior to performing MRD testing, and pathologists must rely on a morphological assessment of the BM aspirate to decide whether to pursue MRD testing. As a result, there is a large proportion of patients for whom MRD testing reveals a PC clone of significant size, far beyond the MRD test’s purpose. In this study, we examined the utility of screening FCM in assessing the need for MRD testing in a cohort of patients treated for MM and plasma cell leukemia (PCL).

The study was approved by the Institutional Review Board at Mayo Clinic, Rochester. We reviewed clinical and laboratory records of patients who underwent FCM testing for a PC malignancy from July 2017 to December 2021. All treated MM or PC leukemia patients with BM PCs <5% by morphology were identified as candidates for potential MRD testing (Fig. 1).

**Fig. 1:**
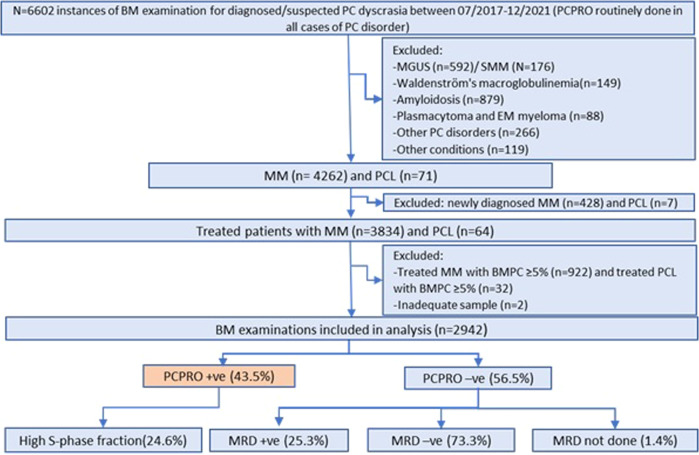
Flow of patients in the study. We initially identified all patients with a plasma cell (PC) disorder who underwent plasma cell proliferation (PCPRO) testing. Treated patients with multiple myeloma (MM) and PC leukemia (PCL) who were considered measurable residual disease (MRD) testing-eligible were identified and the proportion of patients in whom MRD testing was avoided by a positive PCPRO was calculated. BMPC bone marrow plasma cell, EM extramedullary, MGUS monoclonal gammopathy of undetermined significance, SMM smoldering multiple myeloma.

To determine the need for MRD testing, we have used plasma cell proliferation assay (PCPRO) as a screening tool, as previously described [7, 8]. Briefly, PCPRO testing was performed on BM aspirate samples using antibodies to CD19, CD38, CD45, CD138, and kappa and lambda light chains, and 4′,6-diamidino-2-phenylindole (DAPI), a DNA binding dye [8]. 5 × 10^5^ events were acquired per sample using BD FACSCanto™ II (BD Biosciences, Franklin Lakes, NJ) and the analysis was performed by Kaluza software (Beckman Coulter Life Sciences, Indianapolis, IN) [7, 8]. Abnormal clonal PCs were identified using differential expression of surface antigens, kappa-to-lambda ratio, and DAPI staining. Ploidy status was established by calculating the DNA index (DI) which is a ratio of mean fluorescence intensity between G0–G1 peaks of abnormal and normal PCs. The S-phase fraction of abnormal PCs was estimated by dividing the number of abnormal PCs in the S-window by the total number of abnormal PCs after manually gating G0–G1 and G2–M peaks [7]. In prior studies, high plasma cell S-phase fraction has been associated with poor prognosis in plasma cell disorders [7, 9, 10].

MRD FCM was performed by the two-tube 8-color Euroflow method using antibodies to CD19, CD27, CD38, CD45, CD56, CD81, CD117, CD138, and kappa and lambda light chains, as previously described. Bulk lysis and automatic PC identification were performed according to guidelines [5, 6]. Data were analyzed using Infincyt software (Cytognos, Salamanca, Spain).

Among 3753 patients with suspected PC neoplasms, there were 6602 instances where BM aspirates were subjected to PCPRO testing. We identified 2942 analyzable instances in which patients had been treated for MM or PC leukemia and had <5% of PCs in BM aspirate by morphologic differential count. The sequence of testing in the study is depicted in Fig. 1; only samples with a negative PCPRO continued to MRD testing. The median number of events acquired for PCPRO and MRD FCM were 4.94 × 10^5^ (0.2 × 10^5^–4.99 × 10^5^) per sample and 8.51 × 10^6^ (0.32 × 10^6^–9.97 × 10^6^) per sample, respectively, with the corresponding analytical sensitivities of 4.4 × 10^−5^ and 2.4 × 10^−6^. PCPRO detected abnormal PC clones in 43.5% of specimens with a median clonal PC burden of 0.1%. High PC proliferation (S-phase fraction ≥2%) was noted in 24.6% of PCPRO-positive cases. Among PCPRO-negative specimens, 25.4% were MRD-positive with a median clonal PC burden of 0.002%, and 73.3% of specimens were MRD-negative. Taken together, these results show that 57.8% of patients had a measurable residual disease, and of these 75% were detected by the screening PCPRO test, with an additional 25% by the NGF MRD testing. Details of PCPRO and MRD FCM results are given in Supplemental Table 1.

Table 1 shows a comparison of analytical and operational characteristics between PCPRO and NGF MRD. The major benefit of performing screening flow cytometry testing is in the management of laboratory time and resources. MRD testing takes about 10 times more antibodies, 2–3 times more instrument time, and 4 times more analysis time than PCPRO. When comparing our workflow with a theoretical workflow in which MRD was performed on all cases without screening, there is a saving of 6% in instrument time and, more importantly, 25% in analysis time; the savings in antibody cost is difficult to assess as the MRD test is currently performed using a kit provided by the manufacturer, while antibodies for PCPRO are procured separately. While not studied here, the advantages of this approach should translate to settings where NGS is used for MRD assessment by avoiding those samples which are clearly positive by PCPRO. In addition, PCPRO measures S-phase, which is an important prognostic factor even in treated plasma cell neoplasms [9, 10]. PCPRO does not evaluate hemodilution, but the significance of hemodilution in samples with a positive result is minimal. An additional benefit of our screening approach is the fast turnaround time and preservation of PCs for FISH and molecular studies which are performed only on PCPRO-positive cases.

**Table 1:** Analytical and operational differences between the screening PCPRO and NGF MRD tests.

	PCPRO	NGF MRD
Target event collection	5 × 10^5^	1 × 10^7^
Maximum sensitivity	4 × 10^−5^	2 × 10^−6^
Observed median sensitivity	4.4 × 10^−5^	2.4 × 10^−6^
Amount of antibody needed	Low	High
Instrument time per patient	6 min	16 min
Analysis time per patient	5 min	20 min
Gating skills needed	Moderate	High
PC S-phase assessment	Yes	No
Hemodilution assessment	No	Yes
Immune microenvironment assessment	No	Partial

The principal strengths of our study are the large number of samples we studied and the expertise at our institution for the PC proliferation assay [11–15]. However, the technique is not commonly used and independent validation of this assay or a similar approach is necessary. As described earlier, the steps and reagents involved in PCPRO testing are simple and easy to incorporate in any hematopathology lab with FCM expertise.

To the best of our knowledge, this is the first study to show the utility of a screening FCM to avoid unnecessary MRD testing in patients with MM and PC leukemia. Using PCPRO as a screening test provides additional prognostic information.

## Supplementary information

Data supplement

## Data Availability

The datasets generated during and/or analyzed during the current study are available from the corresponding author on reasonable request.
