# Supplementary material for: Utility of flow cytometry screening before MRD testing in multiple myeloma
Source: Blood Cancer J. 2023 Apr 20;13(1):55. doi: 10.1038/s41408-023-00832-8 (PMC10119092; doi:10.1038/s41408-023-00832-8)
Supplement: Supplementary file 1 — Data supplement [file 41408_2023_832_MOESM1_ESM.docx]

| **Supplemental Table 1-** Characteristics of PCPRO and MRD flow cytometry evaluation of samples | |
| --- | --- |
| **Characteristics** | **Summary measures, n (%)** |
| PCPRO non-aggregate events, median(range) | 4.94x10^5^(0.2x10^5^- 4.99x10^5^) |
| **PCPRO +ve** | **1279 (43.5)** |
| Monoclonal plasma cells (%), median(range) | 0.1 (0.001- 21.3) |
| kappa | 867 (67.8) |
| lambda | 398 (31.1) |
| Biclonal | 7 (0.5) |
| Light chain negative | 7 (0.5) |
| S-phase fraction ≥2% | 315 (24.6) |
| S-phase fraction <2% | 423 (33.1) |
| S-phase fraction cannot be calculated | 541 (42.3) |
| Diploid | 413 (32.3) |
| Hyperdiploid | 533 (41.7) |
| Hypodiploid | 99 (7.7) |
| Tetraploid | 48 (3.7) |
| Multiple clones with different DNA index | 21 (1.6) |
| Ploidy cannot be estimated | 165 (12.9) |
| **PCPRO –ve** | **1663 (56.5)** |
| MRD non-aggregate events, median (range) | 8.51x 10^6^ (0.32x 10^6^-9.97x10^6^) |
| MRD +ve | 421 (25.3) |
| MRD (%), median (range) | 0.002 (0.0001- 0.2921) |
| MRD –ve | 1219 (73.3) |
| MRD not done | 23 (1.4) |
